# Wessex Branch of Association of Clinical Pathologists, Bath

**Published:** 1987-11

**Authors:** 


### Wessex Branch of Association of Clinical Pathologists

### Meeting at Bath Postgraduate Medical Centre, November 18th 1986

About fifty members from the south west of England attended a joint clinical pathology meeting held by the Wessex branch of the Association of Clinical Pathologists. The President of the Association of Clinical Pathologists Dr H B Goodall attended, and he reminded the local branch that they had provided two presidents in the last twenty years in Dr G. K. McGowan and Dr A. C. Hunt. In addition, Dr B. Murphy is the current Chairman of Council of the Association of Clinical Pathologists. During the course of the scientific session the following papers were presented.

### CONGENITAL INFECTIONS

**M. Jenkins**
**Southmead Hospital, Bristol**

Up to 15% of all pregnancies are complicated by maternal infections and congenital infections are not rare. Organisms may reach the foetus from the mother by two main routes: Haematogenous spread via the placenta or as an ascending infection usually associated with ruptured membranes. Examination of the placenta and membranes will usually indicate the mode of infection.

At Southmead Hospital all late abortions and 63% of stillbirths and neonatal deaths have autopsies. Within the last year two rare examples of congenital infection have been identified. The first was a case of congenital parvovirus infection where pregnancy was complicated at 16 weeks by a maternal "flu-like" illness followed at 26 weeks by the spontaneous delivery of a hydropic abortus. Maternal serology showed recent parvovirus infection and parvovirus inclusions were present in all the major organs and the placenta. This is the first fully documented case of congenital parvovirus infection.

The second case was an example of congenital syphilis in an infant born at 30 weeks gestation and dying 6 hours later. The major organs showed characteristic histological changes and spirochaetes were demonstrated with silver stains.

A thorough examination of all abortions, stillbirths and neonatal deaths will identify most congenital infections and can be a rewarding exercise for the pathologist, providing useful information for the clinician and parents.

### MICROBIOLOGICAL AND HISTOLOGICAL STUDIES ON THE DIFFERENCES IN VIRULENCE AMONGST LISTERIA GENOSPECIES IN INBRED MICE

**A. P. MacGowan[1], T. Mainou-Fowler[2], R. Postlethwaite[3].**
**[1]Department of Medical Microbiology, Southmead Hospital, Bristol**
**[2]Department of Surgery, University of Aberdeen, Aberdeen**
**[3]Department of Bacteriology, University of Aberdeen, Aberdeen**

DNA homology studies have defined five genospecies amongst the serovars of *Listeria*. These are *Listeria monocytogenes*, a human and animal pathogen, *L. ivanovi*, a sheep pathogen, and *L. innocua*, *L. seelingeri*, *L. welshimeri* which are non-pathogenic. Mean lethal doses, ($LD_{50}$) for several representative strains of these genospecies were established in C57B1/6, resistant, and BALB/C, susceptible, inbred mice. $LD_{50}$s for non-pathogenic genospecies were 100–1000 times greater than those for the pathogenic genospecies and the $LD_{50}$s for *L. ivanovi* was 10 times more than that of *L. monocytogenes*. All genospecies had higher $LD_{50}$s in C57B1/6 mice than BALB/C. Bacterial growth kinetic studies of the five genospecies in murine livers revealed that with non-pathogenic strains there was a decline in bacteria isolated from the liver after inoculation to 72 hrs. In contrast the pathogenic *Listeria* genospecies increased in numbers by 1000 times from 6 hrs to 44 hrs post-infection (pi) and then declined. Histological evaluation of livers over the time course of infection revealed a diffuse increase in polymorphonuclear leukocytes (PMNs) in liver sinusoids at 6 hrs with all genospecies. In *L. ivanovi* and *L. monocytogenes* at 21 hrs pi there were numerous microabscesses distributed throughout the liver while in *L. innocua* and *L. welshimeri* infection no histological changes were apparent. At 72 hrs lesions due to the pathogenic genospecies had a significant number of macrophages and lymphocytes within them and by 9 days pi were granulomatous. In *L. seelingeri*, *L. welshimeri* and *L. ivanovi* no histological changes were noted at 9 days pi.

### PEDUNCULATED HEPATOCELLULAR CARCINOMA
### Is it an entity?

**K. James**
**Royal Devon and Exeter Hospital, Exeter**

Hepatocellular carcinoma is common in Africa and South-East Asia but uncommon in western Europe. In general, these tumours have a poor prognosis. Three variants have a better prognosis, these being fibrolamellar, encapsulated and the recently described pedunculated hepatocellular carcinoma. Most cases of the latter have been reported from Japan but two cases have been encountered recently in Devon.

The first case was a 65 year old female with a six month history of discomfort in the right upper abdomen. A pedunculated hepatocellular carcinoma was resected from an otherwise normal liver. A postoperative alpha-fetoprotein was moderately elevated. Hepatitis B virus markers were negative. The patient is alive and well two years after surgery.

The second case was a 63 year old male with a four year history of intermittent right sided abdominal pain. A pedunculated hepatocellular carcinoma was removed from an otherwise normal liver. Alpha-fetoprotein and hepatitis B virus markers were not done. The patient remains well five years after the onset of symptoms despite a biopsy proven metastasis in the left humerus.

These cases were reviewed with the 30 previously reported. The tumours are histologically and immunohistochemically identical to other hepatocellular carcinomas although fewer are associated with cirrhosis or hepatitis B surface antigen. Our two cases are similar to those previously reported. It is proposed that the good prognosis of these tumours is as a result of their origin in accessory lobes of the liver.

### ENDOCRINE DIFFERENTIATION OF EXTRAPULMONARY SMALL CELL CARCINOMA DEMONSTRATED BY IMMUNOHISTOCHEMISTRY USING ANTIBODIES TO PGP 9.5, NEURON-SPECIFIC ENOLASE AND THE C-FLANKING PEPTIDE OF HUMAN PRO-BOMBESIN

**D. R. Springall, N. B. N. Ibrahim, J. Rode, Mandy S. Sharpe, S. R. Bloom and Julia M. Polak**
**Frenchay Hospital, Bristol and Royal Postgraduate Medical School, London**

Several recent studies have confirmed the endocrine nature of small cell carcinoma of the lung. In extra-pulmonary sites, small cell "undifferentiated" carcinomas have classical morphological features similar to their pulmonary counterpart. We therefore investigated using immunocytochemistry, the possibility that the non-pulmonary neoplasms may also be endocrine in nature. Sections of 29 small cell carcinomas from oesophagus, stomach, larynx, colon, and urinary bladder were immunostained using antisera to protein gene product 9.5 (PGP 9.5), neuron-specific enolase (NSE), cytokeratin, leucocyte common antigen and peptides including bombesin, the C-flanking peptide of human pro-bombesin, andrenocorticotrophic hormone, neurotensin, calcitonin and pancreatic polypeptide. All the tumours showed immunoreactivity for at least one of the two general endocrine markers PGP 9.5 and NSE. All were positive for cytokeratin and negative for leucocyte common antigen. Of the regulatory peptides, immunoreactivity was obtained with antisera to bombesin (one case), the C-flanking peptide of human pro-bombesin (19 cases), adrenocorticotrophic hormone (one case) and calcitonin (three cases). No PGP 9.5−, NSE−, or peptide-like immunoreactivity was detected in 25 control tumours from similar sites, including lymphomas and poorly differentiated tumours. These results suggest that non-pulmonary small cell carcinoma has an endocrine character. Our study has also shown that the C-flanking type of pro-bombesin is a better marker than bombesin.

### THE EFFECT OF HEAT TREATMENT OF SERUM UPON ROUTINE BIOCHEMIAL ANALYSES

**A. A. McConnell**
**Southmead Hospital, Bristol**

The continuing rise in the number of patients suffering from AIDS poses a problem for chemical pathology laboratories, since blood and blood products are the main mechanism for transmission of this disease. The HIV virus, the infective agent in AIDS, has been reported to be inactivated by heating at 56 centigrade for 30 minutes.

The effect of such treatment (in the case of serum and of plasma) upon the commonly measured biochemical parameters has been studied, at Southmead and in several other laboratories. It is generally agreed that serum is to be preferred to plasma and does not require centrifugation after heating. Most biochemical analytes show no change of clinical significance, with the exception of Alkaline Phosphatase, Creatinine Kinase both of which are largely inactivated, and of Acid Phosphatase and Alanine Transaminase, which show decreases of the order of 40% and 60% respectively. LDH may be of some clinical value provided the differing heat sensitivity of the various isoenzymes is remembered, and Gamma-GT may prove to be of value in the light of continuing work. For Aspartate Transaminase, Lactate Dehydrogenase, Amylase, and Gamma gt it has been shown that the decrease on heating is independent of the starting enzyme level.

A note of caution must be sounded for analytes with values outside normal ranges unless and until such abnormal levels are investigated, especially in patients with renal failure, because of the presence of uraemic toxins.

In serum, salicylate and paracetamol levels show clinically insignificant changes although the number of paracetamol samples studied at, or near, the treatment line needs to be increased.

### CAMPYLOBACTER PYLORIDIS—A NEW PATHOGEN?

**Cliodna A. M. McNulty**
**Public Health Laboratory, Gloucester**

These organisms were first seen on the gastric mucosa of man at the turn of the century. Their significance was ignored until successfully cultured by Marshall in 1982. Koch's three postulates need to be proven before a particular micro-organism can be said to cause a disease. The organism must be found in all cases of the disease and its distribution should be in accordance with the lesions observed; it should be cultured outside the body of the host in pure culture for several generations and should reproduce the disease on inoculation into a susceptible animal. *Campylobacter pyloridis* is found overlying the gastric mucosa, in the gastric crypts and in the mucus layer which protects it from gastric acid. Infection is associated with a characteristic histological appearance of the gastric mucosa. There is a polymorphonuclear and mononuclear infiltrate and the surface longitudinal cells are shortened with evidence of increased nuclear activity and a reduced mucin content. In a Gloucester study (where there are direct general practitioner referrals) *C. pyloridis* was found in 41% of patients. 91% of patients with *C. pyloridis* had histologically proven gastritis, conversely only 6% of the 86 patients with normal gastric mucosa had organisms present. In human volunteer studies ingestion of a suspension containing *C. pyloridis* was followed by the development of severe dyspepsia associated with histologically confirmed gastritis. In our treatment trial, completed in Birmingham, bismuth subsalicylate cleared 15 of 18 patients of *C. pyloridis*. Clearance was highly associated with resolution of gastritis, improvement in endoscopic appearance and improvement of heartburn. *C. pyloridis* infection can be diagnosed by microaerobic culture, the biopsy urease test, histopathology or by serological techniques. Koch's postulates have been proven and the evidence in favour of *C. pyloridis* being important in the aetiology of upper gastrointestinal disease is now very strong. Accordingly the organism has aroused great interest amongst gastroenterologists who are now undertaking further treatment trials. Their results will be awaited with great interest.

### MUCOCUTANEOUS MANIFESTATIONS OF AMYLOID

**Helena M Daly**
**Royal Cornwall Hospital, Truro, Cornwall**

Three men were referred for investigation of mucocutaneous purpura. In each case amyloid was diagnosed clinically and confirmed by biopsy of clinically involved tissue. Two patients had underlying myeloma.

Patient 1. (45 years) had an 18 month history of periorbital purpura and a rash on the shoulder. Examination revealed periorbital and oral mucosal purpura, subconjunctival haemorrhage, cutaneous purpura and proteinuria. Lambda light chains were detected in serum and urine, periorbital skin biopsy confirmed amyloid and bone marrow revealed myeloma.

Patient 2. (50 years) had a two year history of extensive oral mucosal purpura and recent onset severe dyspnoea. Biopsy of oral mucosa showed non-specific change only. Examination revealed extensive buccal mucosal and facial purpura, macroglossia and proteinuria. Kappa light chains were present in serum and urine and review of original biopsy with Congo Red stain and polarised light microscopy confirmed amyloid infiltration. Bone marrow was normal.

Patient 3. (72 years) was referred by a dermatologist with periorbital oral mucosal and digital cutaneous purpura. Amyloid was suspected but rectal biopsy was normal. An IgA paraprotein with immunoparesis was detected. Bone marrow showed myeloma and further biopsy of clinically involved skin revealed amyloid.

Each patient received melphalan and prednisolone. Patient 1 is alive two years later with recent symptomatic restrictive cardiomyopathy. Patient 2 died after four months of congestive cardiac failure due to restrictive cardiomyopathy. Post mortem revealed widespread deposition of amyloid. Patient 3 is alive nine months after diagnosis with persistent cutaneous purpura but disappearance of IgA paraprotein. In each case there was a long delay in diagnosis. This was due to failure to recognise clinical signs, unhelpful biopsy results because the clinical information was not provided and selection of an uninvolved biopsy site when a clinically involved area was available.

Amyloid is notoriously difficult to treat. Early treatment directed at the B cells producing the amyloidogenic protein may prevent further deposition.

